# Research Progress of Methane Membrane Separation Technology

**DOI:** 10.3390/membranes16040119

**Published:** 2026-03-28

**Authors:** Xiujuan Feng, Haoyu Zhang, Haotong Guo, Chuhao Huang, Yiwen Fu, Shuqi Wang, Jing Yang, Jie Li, Yankun Ma

**Affiliations:** 1 State Key Laboratory of Deep Coal Safety Mining and Environmental Protection, Anhui University of Science and Technology, Huainan 232001, Chinayj25290@163.com (J.Y.); 2 Key Laboratory of Industrial Dust Control and Occupational Health, Ministry of Education, Anhui University of Science and Technology, Huainan 232001, China; 3 School of Earth and Environment, Anhui University of Science and Technology, Huainan 232001, China; 4 School of Economics and Management, Anhui University of Science and Technology, Huainan 232001, China; 5 School of Safety Engineering, Anhui University of Science and Technology, Huainan 232001, China; 6 School of Marxism, Anhui University of Science and Technology, Huainan 232001, China

**Keywords:** methane, membrane technology, methane purification, polymer membranes, inorganic membranes, metal organic frameworks

## Abstract

Membrane technology demonstrates broad prospects in the field of methane capture and purification due to its high efficiency and low energy consumption characteristics. This paper systematically reviews the research progress in membrane technology for methane separation in recent years, focusing on the design and optimization of membrane material systems, in-depth analysis of mass transfer mechanisms, and practical applications in areas such as biogas upgrading and natural gas decarbonization. Researchers have significantly enhanced membrane separation performance for CO_2_/CH_4_, CH_4_/N_2_, and other systems by developing novel material systems such as polymer membranes, inorganic membranes, and mixed matrix membranes (MMMs), combined with strategies like pore structure regulation, interface optimization, and functionalization. Although membrane technology has shown good economic feasibility and application potential in some scenarios, challenges such as long-term material stability, anti-plasticization capability, and large-scale manufacturing remain the main current obstacles. Future research should further focus on the development of novel membrane materials, process integration optimization, and intelligent process control to promote a greater role for membrane technology in the efficient utilization of methane resources and energy structure transformation.

## 1. Introduction

Methane, as a clean fossil fuel, generates relatively less CO_2_ and pollutants during combustion and is regarded as a crucial bridge in transitioning the energy structure from high-carbon to low-carbon [1]. Conventional separation technologies such as cryogenic distillation, amine absorption, and pressure swing adsorption [2,3] suffer from issues like high energy consumption, complex processes, and challenges in scale-up. In contrast, membrane separation technology has attracted widespread attention in the field of gas separation due to its advantages of high throughput, low energy consumption, and ease of scaling up [4,5,6]. However, traditional membrane materials are limited in selectivity, making it difficult to achieve high-precision separation [7]. Furthermore, their core deficiencies when separating methane (especially from waste gases) lie in their high susceptibility to fouling leading to performance decay, and often excessively high energy consumption and costs when processing low-concentration methane, lacking economic viability [8,9]. In recent years, significant progress has been made by domestic and international scholars in the development of high-performance membrane materials, research on mass transfer mechanisms, and assessment of process feasibility [10]. Internationally, research teams from the United States, Europe, and Japan have led advancements in mixed matrix membranes by incorporating porous fillers such as zeolites and metal–organic frameworks, thereby overcoming the traditional trade-off between selectivity and permeability in polymeric membranes [11]. Driven by the “Dual Carbon Goals,” domestic research has focused on the synthesis of MOF materials and membrane fabrication, aiming to improve the separation performance for systems such as CH_4_/N_2_ and CH_4_/CO_2_ [12,13]. This article concurrently reviews the latest advances, performance bottlenecks, and resolution strategies of three major membrane systems—polymeric, inorganic, and mixed matrix membranes—in methane separation over the past decade. It closely integrates specific application scenarios such as biogas purification, natural gas decarbonization, and unconventional gas treatment, analyzing the suitability and process requirements of different membrane materials, delving into the underlying separation mechanisms, and simultaneously looking forward to future technological development directions. The aim of this review is to provide theoretical reference and technical guidance for promoting a greater role of membrane technology in the efficient utilization of methane resources, optimization of the energy structure, and achievement of carbon neutrality goals.

## 2. Gas Separation Membrane Systems

**Figure 1:**
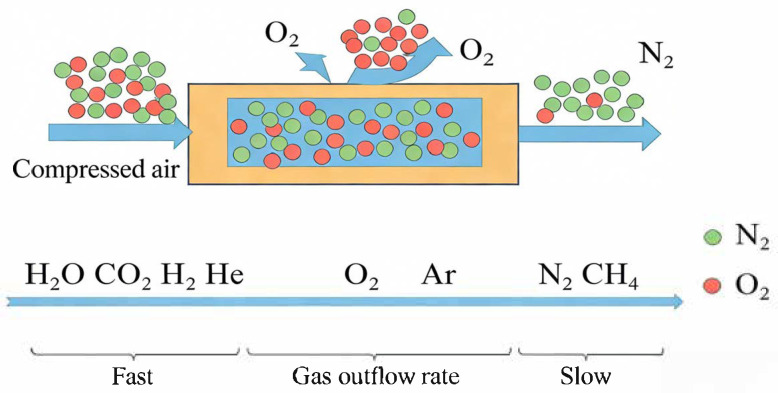
Micropore diffusion mechanism in gas membrane separation.

The transport mechanisms of methane-containing gases within membranes primarily depend on the membrane’s own structure (porous or non-porous/dense), as well as on operating conditions (such as pressure, temperature). Moreover, in practical applications, the predominant mechanisms are typically micropore diffusion and solution–diffusion. The micropore diffusion mechanism primarily applies to porous membrane materials. During operation, when the mean free path of the gas molecules in the mixture is smaller than the pore size of the porous membrane, it facilitates the separation process. Due to the fact that the extent of interaction between the gas molecules and the porous medium is constrained by the pore size and internal surface properties of the porous membrane material, the transport behavior exhibits distinct characteristics during the separation of gas mixtures. The underlying principle is illustrated in Figure 1, using air separation as an example [14].

**Figure 2:**
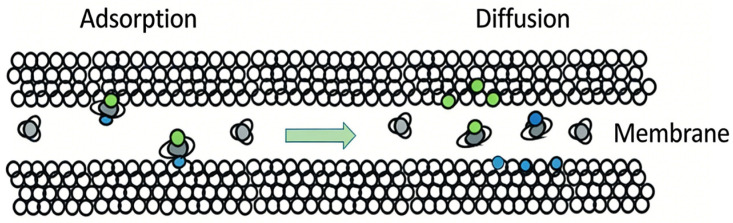
Dissolution diffusion mechanism membrane separation.

The separation process of gas mixtures through non-porous membranes (dense membranes) can be explained by the solution–diffusion mechanism. As illustrated in Figure 2, the adsorption–dissolution and desorption of gases at the membrane surface can quickly reach equilibrium. However, due to the properties of the non-porous membrane material, the time required for gases to permeate through the membrane under permeation–diffusion is relatively long. Therefore, diffusion of gases within the membrane becomes the rate-limiting step in the separation process.

Gas separation membrane technology is a core method for methane capture and purification. Based on material type, it is mainly divided into three categories: polymeric membranes, inorganic membranes, and mixed matrix membranes. Overall, current research is dedicated to breaking the trade-off relationship between permeability and selectivity inherent in traditional membranes through material innovation and structural optimization to meet the needs of different methane separation scenarios.

### 2.1. Polymeric Membranes

Polymer membranes are continuous thin films made from organic polymer materials through methods such as phase inversion, coating, and stretching [15,16]. Their separation mechanism is primarily based on the “solution-diffusion” model, where gas or liquid molecules first dissolve into the membrane material, then diffuse through the membrane driven by concentration or pressure gradients, and finally desorb on the other side.

Due to their mature fabrication processes, good mechanical properties, and low cost, polymer membranes currently dominate the gas separation membrane market [17]. Polymer membrane materials used for methane separation primarily include polyimide (PI), polysulfone (PSF), cellulose acetate (CA), etc. These materials can be fabricated into asymmetric or composite membranes via solution casting or phase inversion methods, with their separation mechanisms primarily following the “solution-diffusion” model [18]. The performance of polymer membranes is significantly influenced by their chemical structure. For instance, polyimides containing rigid segments [19] and bulky side groups typically exhibit higher selectivity and better anti-plasticization resistance, but may have relatively lower gas permeability. A major challenge for polymer membranes is the well-known “upper bound trade-off” (Robeson Upper Bound), where permeability and selectivity often have an inverse relationship. Furthermore, in high-pressure environments or gases containing plasticizers like heavy hydrocarbons or CO_2_, polymer membranes can undergo plasticization, leading to decreased selectivity.

**Figure 3:**
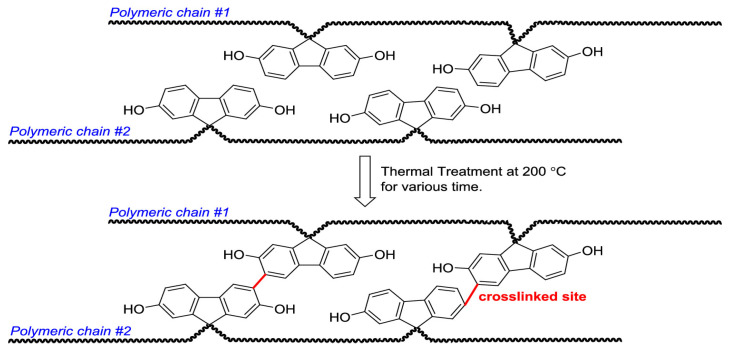
The thermally cross-linked structure of the 6FDA-Durene/Cardo(OH)(3:1) copolymer imide segment prepared in the study [20].

Despite the trade-off challenge, the development of novel polymer materials continues. For example, Ali Hayek et al. [20] reported a thermally cross-linked membrane based on a 6FDA-Durene/CARDO(OH) (3:1) copolyimide, aimed at enhancing its performance in high-pressure mixed-gas separation. A CARDO(OH) monomer containing hydroxyl groups was synthesized to fabricate the thermally cross-linkable copolyimide membrane, which was then subjected to cross-linking treatment at 200 °C for varying durations (24–96 h). The segmental thermal cross-linking structure is shown in Figure 3. The experiments were conducted using pure gases (He, N_2_, CH_4_, CO_2_) and a mixed gas (approximately 28% CO_2_, 62% CH_4_, 10% N_2_) under high-pressure (up to 62.0 bar) and elevated temperature (25–55 °C) conditions to simulate natural gas purification environments. The results indicated that after 96 h of thermal cross-linking, the membrane exhibited a mixed-gas CO_2_ permeability coefficient of 219 Barrer and a CO_2_/CH_4_ selectivity of 26.1 at 34.5 bar and 45 °C, with no signs of plasticization observed even at pressures as high as 62.0 bar. Following cross-linking, the membranes showed reduced solubility and decreased fractional free volume, leading to a decline in gas permeability but a notable improvement in selectivity. These characteristics demonstrate excellent stability and potential for industrial applications [21].

Comesaña-Gándara et al. [22] designed and synthesized a series of ultrapermeable polymers of intrinsic microporosity (PIMs) based on benzotriptycene building blocks. These polymers exhibit BET surface areas of 848–1034 m^2^/g and CO_2_ permeabilities of 21,000–53,000 Barrer, while maintaining excellent CO_2_/CH_4_ and CO_2_/N_2_ selectivities. All data points significantly surpass the 2008 Robeson upper bounds and display a linear correlation, thereby redefining the performance limits for these two critical gas pairs, with the new CO_2_/CH_4_ upper bound showing approximately 2.5-fold higher selectivity than the 2008 bound. This work establishes a new benchmark for the molecular design of polymer membranes. Lai et al. [23] synthesized a class of hydrocarbon ladder polymers with three-dimensional contorted backbones via catalytic arene-norbornene annulation (CANAL) polymerization. Unlike conventional PIMs, these polymers exhibit anomalous aging behavior: the diffusion coefficients of larger gases (CH_4_, N_2_) decrease significantly more than those of smaller gases (H_2_, CO_2_) during physical aging, leading to substantial enhancements in H_2_/CH_4_ and CO_2_/CH_4_ selectivities while maintaining relatively high permeability. For instance, after 158 days of aging, CANAL-Me-DHP achieved a CO_2_/CH_4_ selectivity of 68 and an H_2_/CH_4_ selectivity of 621. In mixed-gas tests, it maintained a selectivity > 35 under a CO_2_ partial pressure of 14 bar, far exceeding the 2018 mixed-gas upper bound. This work reveals a new approach for optimizing transport bottlenecks through backbone configuration tuning.

Subsequently, to improve membrane selectivity in CH_4_/N_2_ systems, Buonomenula et al. [24] prepared nanostructured gas separation membranes based on polystyrene butadiene styrene triblock copolymer (SBS). They used two SBS materials with different styrene contents: SBS28 (28 wt% styrene) and SBS21 (21 wt% styrene). The membranes were pure polymer systems, with nanostructure control primarily achieved by selecting different solvents (toluene or chloroform) and concentrations. The study tested the separation performance for CO_2_, N_2_, and CH_4_ gases, focusing on the separation of CH_4_/N_2_ and CO_2_/N_2_ pairs. Among them, the SBS21 membrane prepared in toluene exhibited the highest ideal CH_4_/N_2_ selectivity (7.2) and good CO_2_ permeability (289 Barrer). This performance enhancement was attributed to the formation of ordered columnar structures perpendicular to the membrane surface by the polystyrene hard segments. Yang et al. [25] prepared cross-linked membranes by reacting SBS with poly(dimethylsiloxane-co-methylhydrosiloxane) (PDMS-co-PMHS) via hydrosilylation. Under the same cross-linking degree, this method allowed for the introduction of more Si–O bonds into the cross-linked membrane. PDMS-co-PMHS was introduced as a cross-linker through chemical cross-linking, with its content ranging from 10 wt% to 70 wt%. The study focused on the separation of the CH_4_/N_2_ system, aiming to enhance methane permeability in coalbed methane. Experiments showed that as the PDMS-co-PMHS content increased, the CH_4_ permeability of the cross-linked membrane significantly increased (reaching up to 443.6 Barrer, over 10 times that of the pure SBS membrane), while the CH_4_/N_2_ selectivity remained around 3.1. This system further increased CH_4_ permeability at 55 °C, demonstrating potential application value in CH_4_/N_2_ separation. Zheng et al. [26] synthesized two ladder polymers, P1 and P2, based on pentiptycene using a stoichiometric imbalance-promoted step-growth polymerization method based on a self-accelerating Diels–Alder reaction under mild, catalyst-free conditions. The polymers possessed rigid, twisted backbones and exhibited intrinsic microporosity (BET surface area > 618 m^2^/g, pore size 0.4–1.4 nm). Self-supported films were prepared by dissolving the polymers in NMP (N-methyl-2-pyrrolidone, a high-boiling polar solvent) using a solvent evaporation method. Gas permeation tests showed that P1 membrane exhibited excellent separation performance for CO_2_/CH_4_. After aging for 14 days, its CO_2_ permeability was 220 Barrer and CO_2_/CH_4_ selectivity reached 21.41, approaching or exceeding the 2008 Robeson upper bound. In contrast, the P2 membrane, incorporating flexible isopropoxy side chains, showed decreased performance due to reduced free volume. The experiments indicated that a rigid ladder structure is beneficial for improving gas separation performance, although physical aging can lead to decreased permeability. This work provides a new perspective for enhancing the gas separation performance of PIMs membranes through backbone rigidity design. Based on the above data, it can be concluded that while the separation performance of polymer membranes is limited, their readily available raw materials and easy processability make them highly suitable as a matrix for mixed matrix membranes.

In summary, to overcome these bottlenecks, current research should primarily focus on the following improvement directions: first, enhancing chain rigidity through molecular design, such as introducing rigid segments (e.g., in polyimides) or bulky side groups, to simultaneously improve separation precision and anti-plasticization capability; second, developing entirely new polymers with specific cavity structures to optimize gas molecule diffusion pathways; third, using polymers as the continuous phase in mixed matrix membranes to synergistically enhance performance by incorporating porous fillers (e.g., MOFs, zeolites), with the key being solving the interfacial compatibility issue between fillers and the polymer matrix to avoid interfacial defects.

### 2.2. Inorganic Membranes

Inorganic membranes, as high-performance separation media prepared from materials like ceramics, metals, and carbon, are increasingly studied for applications in harsh environments. Their core advantages lie in excellent stability, with tolerance to high temperatures, strong acids/bases, and organic solvents. They also possess high strength, long lifespan, excellent anti-fouling properties, and ease of cleaning, allowing for efficient regeneration through rigorous physicochemical means. However, the technology still faces bottlenecks limiting its widespread application: high manufacturing costs, inherent material brittleness, difficulties in large-area defect-free fabrication and reliable high-temperature sealing, and relatively low functional flexibility.

Within the field of inorganic membranes, those used for gas separation mainly include dense membranes and porous membranes, categorized based on pore structure and separation mechanism. In methane separation, research focuses on porous inorganic membranes [27] with precise sieving functions, aiming to achieve efficient separation of systems like CH_4_/N_2_ and CO_2_/CH_4_ through pore size control. Among them, carbon molecular sieve (CMS) membranes and zeolite molecular sieve membranes are two of the most representative materials [28,29].

Molecular sieve membranes exhibit unique advantages in difficult-to-separate systems like CH_4_/N_2_ due to their ordered pore structures and size exclusion effects, but their complex fabrication processes and high costs limit large-scale application. Therefore, Cai et al. [30] studied the preparation of carbon molecular sieve membranes (CMSMs) based on thermally rearranged (TR) precursors from hydroxyl-containing copolyimides (6FDA/ODA/6FAP), with molar ratios of 6FAP to ODA being 0:100 (HPI-0), 30:70 (HPI-0.3), and 50:50 (HPI-0.5), respectively. The base polymer was 6FDA-ODA-6FAP copolyimide. The study tested the separation performance for H_2_, CO_2_, N_2_, and CH_4_, focusing on the separation effects of H_2_/CH_4_ and CO_2_/CH_4_. As the 6FAP content increased, the CMS membranes exhibited more ordered structures and narrower pore sizes. Among them, the TR-CMS-0.5 membrane showed the best methane sieving performance, with H_2_/CH_4_ selectivity reaching 610 and CO_2_/CH_4_ selectivity reaching 143, both surpassing the 2019 Robeson upper bound. Moreover, its CO_2_/CH_4_ separation performance in mixed-gas tests also significantly exceeded the 2018 mixed-gas upper bound, demonstrating excellent methane sieving capability and potential industrial application value. To improve the gas separation performance of carbon molecular sieve membranes, Guo et al. [31] developed a simple strategy combining ionic liquids with porous carbon molecular sieve membranes. Experiments involved preparing carbon molecular sieve (CMS) membranes by pyrolyzing Matrimid^®^ 5218 polyimide (Huntsman Advance Materials Americans Inc, The Woodlands, TX, USA), followed by spin-coating ionic liquid (IL) as a functional filler onto the membrane surface at concentrations ranging from 0.001 to 100 wt%, forming IL/CMS composite membranes. Their research indicated that the ionic liquid did not penetrate into the membrane pores but formed an ultrathin layer on the surface, acting like a “smart gate” to regulate gas entry. The fabricated composite membrane exhibited excellent separation performance for CO_2_/N_2_ mixed gas, with CO_2_ permeability exceeding 600 Barrer and CO_2_/N_2_ selectivity exceeding 50, breaking the traditional Robeson upper bound. Further molecular dynamics simulation results confirmed that this molecularly thick ionic liquid layer indeed produced an effective “gating” effect. Under IL regulation, high CO_2_/CH_4_ selectivity could also be achieved, further expanding its application potential in carbon capture and natural gas purification. Additionally, Yang et al. [32] successfully synthesized nano-K-chabazite zeolite aggregates (NCHA) with annular hierarchical pore structures using a seed-assisted method. Using K^+^ as the balancing ion and iteratively using previous generation crystals as seeds without organic templates, they obtained a stable structure with crystal grain size of about 50 nm and aggregate size of about 500 nm. This material was primarily used for CH_4_/N_2_ separation. Under conditions of 298 K and 1 bar, it showed a high CH_4_ adsorption capacity of 40.12 cm^3^/g and a CH_4_/N_2_ selectivity of 4.7, with significantly enhanced gas diffusion rates. Breakthrough experiments indicated that its separation yield for a CH_4_/N_2_ (50:50) mixture could reach 6939 mL/kg, increasing methane purity to 84%. Its performance surpassed that of traditional zeolite adsorbents, and it has achieved scale-up production at the 130 kg level, demonstrating good prospects for industrial application. Zhou et al. [33] proposed a novel separation strategy based on molecular shape disparity rather than size exclusion to address the challenge of N_2_/CH_4_ separation, where the two molecules have similar kinetic diameters. By partially substituting fumarate with methyl-modified mesaconate linkers in the Zr-fcu-MOF framework, they transformed the original trefoil-shaped pore windows into asymmetric apertures, disrupting the geometric matching with tetrahedral CH_4_ while allowing linear N_2_ to pass through. The optimized Zr-fum_67_-mes_33_-fcu-MOF membrane achieved an N_2_/CH_4_ selectivity > 15 and an N_2_ permeance > 3000 GPU, with stable performance under pressures up to 50 bar and over 150 days of continuous operation. Techno-economic analysis indicated that this membrane could reduce natural gas purification costs by 66–73%.

Molecular sieve membranes exhibit exceptional selectivity in methane separation (especially for systems with similar molecular sizes like CH_4_/N_2_) due to their regular pore structure and precise molecular sieving effect, and they typically possess excellent chemical and thermal stability [34]. However, their development is constrained by complex preparation processes, high costs, and inherent brittleness, posing challenges for large-scale manufacturing and integrated application. Future research should focus on developing low-cost, readily available precursors or green synthesis pathways to reduce costs; improving membrane layer quality and substrate adhesion through technologies like nanocrystal self-assembly and interlayer modification; and combining the excellent performance of inorganic membranes with more economical fabrication and forming processes, which is key to promoting their practical application.

### 2.3. Mixed Matrix Membranes

**Figure 4:**
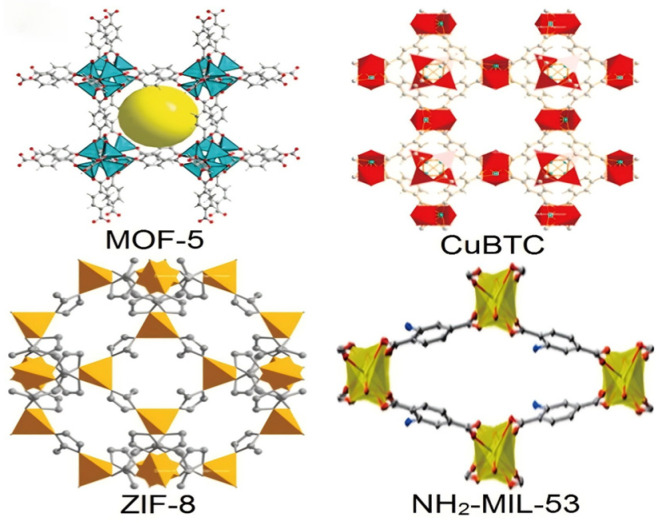
Crystalline structure for some of the MOFs [39].

Mixed matrix membranes are a class of composite materials developed to combine the advantages of polymer membranes and molecular sieve membranes [35]. They are membranes made by uniformly dispersing inorganic fillers (such as molecular sieves, MOFs, carbon molecular sieves, silica, etc.) as the dispersed phase within a polymer continuous phase (matrix). Their separation performance results from the synergistic effect of the polymer matrix and the filler particles [36]. MOFs are an emerging class of porous materials composed of inorganic metal-containing nodes connected by organic coordination bonds. Due to the diversity of metal atoms and linkers, MOFs typically possess a range of unique structural characteristics, such as structural diversity (Figure 4), large specific surface area, and tunable pore structures [37]. These properties make them highly attractive for various applications. Nanoscale molecular sieve membranes composed of porous two-dimensional metal organic nanosheets can achieve high gas flux and high selectivity for gas separation, holding great potential for practical gas separation applications [38].

**Figure 5:**
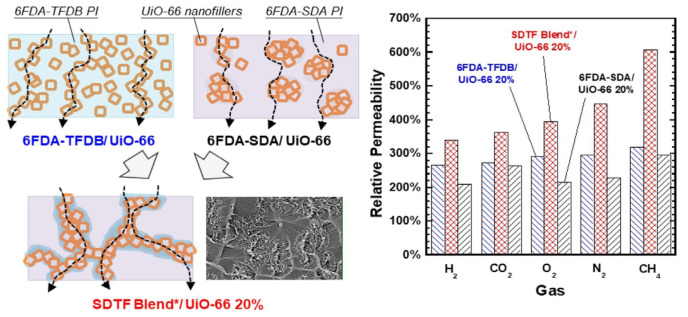
A mixed matrix membrane based on 6FDA-based polyimide and UiO-66 metal organic framework [42].

In the direction of mixed matrix membranes, research focuses on combining porous fillers with a polymer matrix to synergistically enhance separation performance [40]. Overall, MMMs successfully integrate the processability of polymers with the sieving/adsorption selectivity of fillers, becoming an effective pathway to break through traditional performance upper bounds. However, their interfacial compatibility and long-term stability remain core challenges for achieving industrial application [41]. To overcome these issues, Jheng et al. [42] fabricated a mixed matrix membrane (MMM) based on a blend of 6FDA-derived polyimides and the metal–organic framework UiO-66. UiO-66 nanocrystals, synthesized via a solvothermal method, possess excellent gas adsorption properties and a microporous structure. By incorporating UiO-66 into an immiscible blend of two polyimides synthesized from 6FDA—namely 6FDA-SDA and 6FDA-TFDB—an MMM with a co-continuous structure was shown in Figure 5. The “*” in the figure is a symbol used to indicate that the material has undergone post-processing.

**Figure 6:**
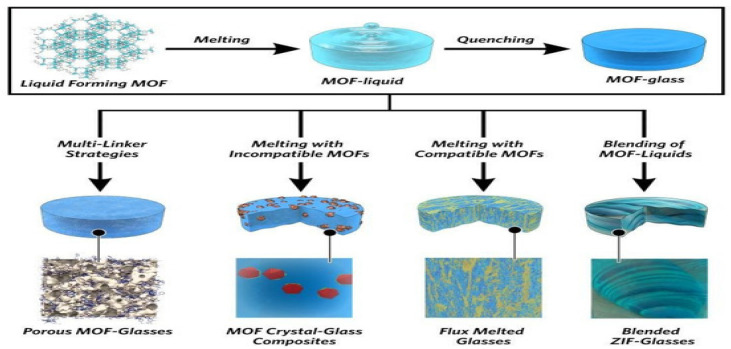
Schematic diagram of the MOF glass fabrication process [44].

Experimental measurements of gas permeability, adsorption, and diffusion revealed that the addition of UiO-66 nanoparticles significantly enhanced gas permeation: for CO_2_, the permeability increased by up to 635%, without compromising gas selectivity. The UiO-66 filler promotes gas adsorption and provides additional pathways for gas diffusion. Wang et al. [43] investigated the incorporation of ZIF-301 metal–organic framework as a filler into 6FDA-DAM polyimide membranes for CO_2_/CH_4_ separation. Experimental results showed that incorporating ZIF-301 into the 6FDA-DAM matrix significantly enhanced both the CO_2_ permeability and the CO_2_/CH_4_ selectivity of the membrane. The modified membrane achieved a CO_2_ permeability of 891 Barrer and a CO_2_/CH_4_ selectivity of 29.3, surpassing the 2008 Robeson upper bound. However, when the ZIF-301 loading reached 30%, the separation performance declined due to filler aggregation and increased rigidity of the polymer matrix. Furthermore, the Bennett team innovatively developed MOF glass membranes [44] by converting crystalline ZIF-62 into an amorphous glassy state through melt-quenching, eliminating grain boundary defects, as shown in Figure 6. This glassy MOF structure eliminates grain boundaries while maintaining short-range order, presenting long-range disorder. Based on these characteristics, MOF glass membranes have become strong candidate materials for high-performance separation membranes [45].

Datta et al. [46] proposed a “three-element synergistic design” strategy to address the challenges of filler agglomeration and interfacial incompatibility in mixed matrix membranes. They tailored a fluorinated MOF (AlFFIVE-1-Ni) with excellent CO_2_/CH_4_ sieving performance into high-aspect-ratio (>25) (001) nanosheets, and achieved in-plane alignment within a polyimide matrix via slow solvent evaporation-induced assembly, successfully obtaining a [001]-oriented mixed matrix membrane. This configuration aligns the 1D channels of the MOF in parallel, forming continuous pathways for gas permeation, and delivers exceptionally high H_2_S/CH_4_ and CO_2_/CH_4_ separation performance under high-pressure mixed-gas conditions. This work provides a generalizable strategy for transforming crystalline porous materials into processable high-performance membranes.

Addressing non-MOF materials, Masor et al. [47] prepared membranes by mixing dried polyetherimide with zeolite 4A filler in N-methylpyrrolidone solvent, followed by stirring, sonication, degassing, doctor-blade coating, and a two-step drying process. The zeolite 4A filler loading ranged from 0 wt% to 20 wt% of the polymer weight. The optimal loading, determined via response surface methodology, was 20 wt%. This membrane was used for CO_2_/CH_4_ separation. Results showed that as zeolite loading increased, CO_2_ permeability significantly increased (>90% higher than the pure polymer membrane), while CH_4_ permeability increased only slightly. Under optimal conditions (20 wt% zeolite, 6 bar pressure), the CO_2_/CH_4_ selectivity reached 3.244, about 45% higher than that of the pure polyetherimide membrane, demonstrating good CO_2_-preferential permeation characteristics. However, permeability to CH_4_ was low, indicating effective CO_2_/CH_4_ separation and enhanced selectivity. Additionally, Laura et al. [48] dissolved three different base polymers—Matrimid 5218 (polyimide), Pebax 1657 MH (polyether block amide), Pebax^®^ 1657 MH was supplied by Arkema (Colombes, France),and a chitosan/polyvinyl alcohol blend (CS:PVA)—in their respective solvents. Porous organic polymer (POP) fillers were added at different loadings. After stirring, sonication, and degassing, membranes were cast, and the solvent was removed via stepwise heating or room temperature evaporation. POP filler loadings ranged from 0 wt% to 32 wt% of the polymer weight, varying with the polymer matrix and POP type (20 wt% in Matrimid, 5–32 wt% in Pebax, 5–10 wt% in CS:PVA). The membranes were used for CO_2_/CH_4_ separation (test gas: 50:50 *v*/*v*% mixture). Results showed that all MMMs improved CO_2_ permeability. Among them, CS:PVA-based MMMs exhibited the best CO_2_/CH_4_ separation selectivity (66.59 for 5 wt% POP3/CS:PVA), significantly outperforming pure polymer membranes. Pebax-based MMMs showed a wide selectivity range (3.98–16.44), while Matrimid-based MMMs maintained high selectivity (~37–42) while also improving CO_2_ permeability. The study noted that the porosity of the filler and the hydrophilicity of the polymer matrix jointly influence separation performance. CS:PVA-based MMMs, due to their good filler compatibility and hydrophilic environment, show potential in CO_2_/CH_4_ separation approaching or even surpassing traditional Pebax membranes, providing a new direction for developing sustainable membrane materials. Tan et al. [49] addressed the long-standing challenge of interfacial incompatibility between zeolite fillers and glassy polymers by selecting a Na-SSZ-39 zeolite with a three-dimensional channel system and high aspect ratio (~12). Through precise control of dispersion and solvent evaporation, they successfully fabricated mixed matrix membranes with zeolite loadings up to 55 wt% and defect-free interfaces. The resulting membrane achieved a CO_2_/CH_4_ selectivity of approximately 423 and a CO_2_ permeability of approximately 8300 Barrer under mixed-gas conditions, outperforming all polymer-based membranes and even most pure zeolite membranes, while maintaining stable performance after 360 days of aging. This work provides an effective paradigm for the preparation of high-loading zeolite-based MMMs.

In summary, mixed matrix membranes aim to break the performance upper bounds of traditional polymer membranes, achieving synergistic improvement in permeability and selectivity. The key to successful MMMs lies in solving the interfacial compatibility issue between fillers and the polymer matrix. Poor interfaces can lead to non-selective interfacial voids (defects) or interfacial blockage, which may instead reduce separation performance. In addition to the aforementioned traditional MOF fillers, a range of novel cutting-edge materials that have emerged in recent years offer more options for the design of MMMs. For example, two-dimensional materials such as graphene oxide (GO) and MXene, with their atomic-level thickness and precisely tunable interlayer channels, have shown great potential in CH_4_/N_2_ sieving. By regulating the interlayer cations or functional groups, their d-spacing can be precisely controlled between 3.4 and 4.0 Å, enabling molecular sieving of N_2_ (3.64 Å) and CH_4_ (3.80 Å), which have similar kinetic diameters [50]. Furthermore, covalent organic frameworks (COFs) have attracted attention in CO_2_/CH_4_ separation due to their low density, high chemical stability, and organically tunable pore environment. Their CO_2_-philic pore surfaces can significantly promote the dissolution and transport of CO_2_ [51]. Of particular note are the breakthroughs in the molecular design of polymers of intrinsic microporosity (PIMs). Researchers have further distorted the polymer backbone by introducing structures such as Troger’s Base or ethanoanthracene, constructing novel PIMs with higher rigidity and enhanced resistance to physical aging. This allows them to maintain high permeability while delaying performance decay [52]. Therefore, future improvements should focus on in-depth interface engineering, enhancing compatibility through surface modification of fillers, optimizing filler dispersion to reduce agglomeration, and actively exploring novel fillers like MOF glasses to fully realize the separation potential of composite materials.

In conclusion, it is noteworthy that with the advancement of materials science, gas separation mechanisms have transcended the traditional solution diffusion and micropore diffusion models. Emerging separation mechanisms are continuously being developed, offering new ideas for breaking the permeability-selectivity trade-off. For instance, the “gating mechanism” utilizes external stimuli (such as pressure or temperature) or intermolecular interactions to achieve reversible opening and closing of pores, thereby precisely regulating the passage of gas molecules; ionic liquid-modified carbon molecular sieve membranes are a typical example. Furthermore, the “facilitated transport mechanism” significantly enhances the solubility and diffusion rate of specific gases (such as CO_2_) in the membrane by introducing carriers (e.g., amine or carboxyl groups) that react reversibly with them. This design principle has been widely applied in fixed-site carrier membranes and some MOF membranes. The core design principle of these mechanisms lies in shifting from relying solely on size sieving to utilizing the dynamic behavior of pores or the chemical affinity of membrane materials to achieve efficient recognition of molecules with similar dimensions.The following is a summary table that presents a comparison of the properties of different materials, as detailed in Table 1.

## 3. Application Status of Membrane Technology in Methane Separation

Membrane technology has demonstrated application potential in the separation of various methane-containing gas mixtures. Its core application scenarios revolve around methane purification and recovery.

### 3.1. Biogas/Landfill Gas Upgrading (CO_2_/CH_4_ Separation)

Biogas (mainly composed of CH_4_ and CO_2_) upgrading is one of the most mature application areas for membrane technology [53,54,55]. The goal is to remove CO_2_ to obtain high-purity biomethane (purity > 95%), which can be injected into the natural gas grid or used as vehicle fuel. Since CO_2_ has a smaller kinetic diameter than CH_4_ and usually higher solubility and diffusivity in polymer membranes, most polymer membranes are selective for the CO_2_/CH_4_ system. MMMs containing MOFs (e.g., ZIF-8) can significantly enhance the separation factor and permeation flux for biogas upgrading, reducing energy consumption and membrane area. Liu Minglong et al. [56] experimentally optimized the membrane biogas upgrading process, addressing issues of temperature drop, plasticization, and fouling. By setting up systems for drying and dehydration, heating, oil-free compressors, and multi-stage filtration, and using SERAN membranes (αCO_2_/CH_4_ = 50) resistant to plasticization, they achieved a product gas with methane content of 96.99% and CO_2_ content reduced to 1.84%, with a methane recovery rate of 96.2%, under feed pressure of 1.35 MPa and temperature of 25–30 °C. This conclusion indicates that process control and component selection effectively mitigated temperature drop, plasticization, and fouling issues. Subsequently, Du et al. [57] prepared a series of polyimide/ionic liquid hybrid membranes by incorporating four imidazole-based ionic liquids (IL_1_–IL_4_) with different alkyl chain lengths into a polyimide (ODA-6FDA) matrix at loadings of 5%, 10%, 15%, and 20%, and systematically investigated their mechanical and gas separation properties. Experimental results showed that the membrane containing 15% of 1-octyl-3-methylimidazolium tetrafluoroborate (IL_3_) exhibited optimal CO_2_/CH_4_ separation performance: a CO_2_ permeability of 16.25 Barrer and a CO_2_/CH_4_ selectivity of 180.55, which is about 2.5 times that of the neat polyimide membrane (73.54) and exceeds the 2008 Robeson upper bound. Meanwhile, the mechanical properties of this membrane were significantly improved, with an elongation at break of 21.09% and a tensile strength of 151 MPa, substantially higher than those of the pure membrane (4.86%, 44 MPa). The study indicates that the introduction of ionic liquids enhances both selectivity and mechanical strength by improving CO_2_ solubility and forming hydrogen-bond interactions, providing a feasible approach for designing high-performance CO_2_/CH_4_ separation membranes. Overall, membrane biogas purification technology has achieved large-scale application. Future development directions should be based on R&D of new membrane materials, coupled processes, and simultaneous removal of multiple impurities.

### 3.2. Natural Gas Decarbonization and Upgrading (CO_2_/CH_4_ Separation)

**Figure 7:**
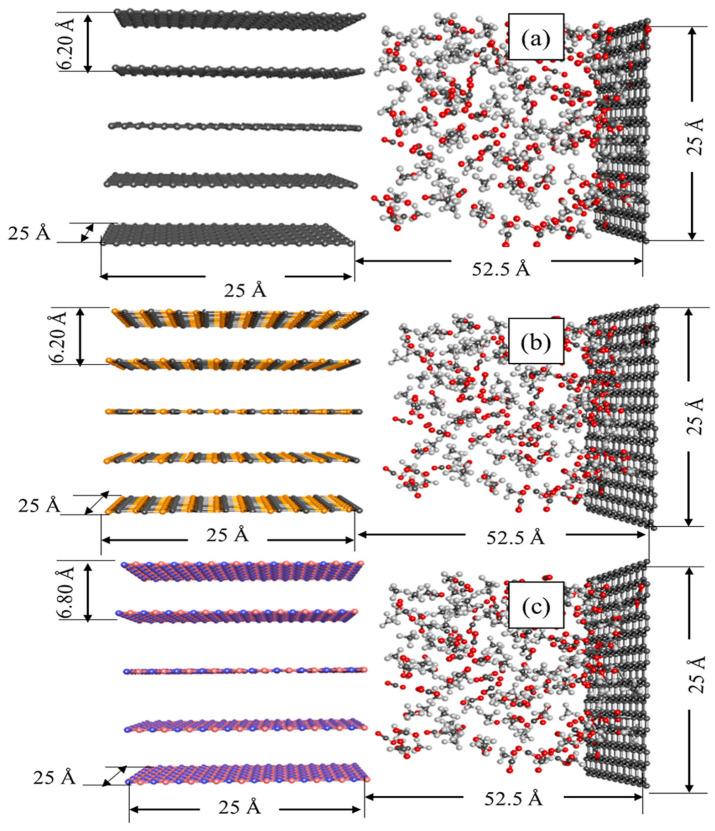
A visual presentation of the simulation box for (**a**) graphene, (**b**) SiC, and (**c**) BN nanochannels [60].

Many oil associated natural gases contain high levels of CO_2_ (sometimes up to over 70%), which needs to be removed before transportation and use. Membrane adsorption technology is particularly suitable for space-constrained scenarios like offshore platforms due to its modularity and small footprint. In this application, membrane materials need to withstand higher pressures (up to 10 MPa or more) and resist corrosion from acidic gases like H_2_S, thus placing higher demands on the membrane’s mechanical strength and chemical stability. Addressing material design issues, Yu et al. [58] systematically studied the separation mechanism of CH_4_/CO_2_ mixtures in various covalent organic frameworks (COFs, e.g., CTF-1, CTF-2, COF-LZU1, COF-5, NUS-2) as membrane materials using molecular simulation methods (GCMC/MD). The study showed that COFs containing oxygen functional groups (e.g., COF-5, NUS-2) exhibited high adsorption selectivity for CO_2_ (e.g., CO_2_/CH_4_ adsorption separation ratio of 17.46 for COF-5) and diffusion selectivity, effectively preferentially adsorbing and hindering CO_2_, thereby enriching CH_4_ in the permeate. Simulation results indicated that separation efficiency was more optimal under conditions of low temperature, relatively high pressure, and high CO_2_ concentration (>40 vol%). Furthermore, the AA stacking mode favored gas diffusion separation. The advantages of these materials for methane decarbonization and upgrading lie in their high selectivity, avoidance of liquid absorbents, and potential for low energy consumption, making them particularly suitable for treating natural gas with high CO_2_ concentration, providing a theoretical design basis for efficient, low-cost membrane separation technology. Xiao Rongge et al. [59] proposed a combined process integrating natural gas membrane separation for decarbonation with LNG(liquefied natural gas) light hydrocarbon recovery. They simulated both the single process and the integrated process using HYSYS software and optimized the integrated process parameters using response surface methodology and genetic algorithms. Research data indicated that the feed gas had a CO_2_ volume fraction of 46.7%, and the LNG composition was mainly CH_4_ (87.97%). The processing scales were 130,000 m^3^/d (decarbonation) and 100 t/h (LNG). Overall, combining membrane separation decarbonation with LNG cold energy utilization can achieve energy consumption reduction and comprehensive resource utilization, demonstrating good industrial integration potential and green process prospects. Furthermore, a research team (Azamat, Alizadeh, Ajalli, Nigeria) [60] constructed three different nanochannel models (graphene, SiC, BN, New York, NY, USA), introduced an equimolar mixture of CH_4_/CO_2_ gas into a simulation box, and performed molecular dynamics simulations at different pressures (100–5000 kPa),as shown in Figure 7. By analyzing key indicators such as gas permeability, density distribution, diffusion coefficients, and interaction energies, they deeply revealed the behavioral differences and intrinsic mechanisms of these three materials in the gas separation process. The research demonstrated that by precisely controlling the material type of the nanochannels (thereby regulating their surface chemistry and polarity), the CH_4_/CO_2_ separation process can be effectively directed. Combining graphene’s high permeability, SiC’s balanced selectivity, and BN’s ultra-high selectivity is an effective strategy for constructing next-generation high-performance hybrid separation membranes. It aims to achieve the dual optimization of permeability and selectivity in gas separation processes through structural design and material combination. This strategy reflects the evolutionary trend from single materials toward multifunctional composite materials.

### 3.3. Unconventional Natural Gas Treatment (N_2_/CH_4_ Separation)

Separating N_2_ from unconventional natural gases with high nitrogen content (e.g., shale gas, coalbed methane) is a more challenging task because CH_4_ and N_2_ have very similar molecular sizes and physical properties. Polymer membranes based on the solution diffusion mechanism generally have low selectivity for this system. Therefore, research in this field focuses on membrane materials with precise sieving functions [61].

In the field of N_2_/CH_4_ separation for unconventional natural gas, Xin et al. [62] prepared an EVA gas separation membrane via the solution casting method and also fabricated Pebax/EVA blend membranes using the same technique. The surface morphology, physical structure, thermal properties, and mechanical properties of these membranes were studied in detail. Testing revealed that the Pebax/EVA (10 wt%) composite membrane exhibited optimal compatibility, with a smooth and dense surface, while the Pebax/EVA (30 wt%) composite membrane showed the lowest crystallinity and a CH_4_ permeability as high as 384.6 Barrer, which is 156.6% higher than that of the pure EVA membrane. The results indicate that blending Pebax with EVA can improve the CH_4_/N_2_ separation performance of the membranes. Among them, the EVA membrane containing 30% Pebax demonstrated the best separation efficiency. It should be noted that increasing temperature enhances gas permeability but reduces selectivity, whereas pressure has a relatively minor impact on separation performance.

Xie et al. [63] successfully fabricated highly compatible mixed matrix membranes (MMMs) by the in situ crystallization of metal–organic frameworks (MOFs, such as CuBTC, ZnBTC, etc.) within a polymer of intrinsic microporosity (PIM-1) via a one-pot room-temperature ball-milling mechanochemical method. They conducted material characterization, gas permeation and separation performance tests, and stability evaluations. The experimental results showed that the MMMs exhibited uniform filler dispersion and tight interfacial adhesion, delivering excellent performance for CH_4_/N_2_ and CO_2_/N_2_ separation. Specifically, the CBP_m_c-35.5 MMM achieved a CH_4_ permeability of 8120 Barrer with a CH_4_/N_2_ selectivity of 6.52, and a CO_2_ permeability exceeding 33,000 Barrer with a CO_2_/N_2_ selectivity of 20–25, representing an improvement of 1–3 orders of magnitude over conventional membranes. Even after 180 days of aging, the membrane retained a CH_4_ permeability of 7260 Barrer and a CH_4_/N_2_ selectivity of 7.11. The membranes also demonstrated good thermal stability, moisture resistance, and universality, providing an efficient and energy-saving new pathway for natural gas purification. Wei et al. [64] reviewed existing technologies for CH_4_/N_2_ separation from low-concentration coalbed methane and research progress on three types of microporous adsorbents: zeolites, metal–organic frameworks (MOFs), and porous carbons. Through experimental methods such as ion exchange and crystal size control, combined with simulation techniques including molecular dynamics (MD) and grand canonical Monte Carlo (GCMC), they identified an optimal pore size range of 0.4–0.7 nm. The modified Ag-ZK-5 zeolite achieved a CH_4_/N_2_ selectivity of 11.8, the Al-Fum MOF reached 17.2, and the ACS-700-2 sulfur-doped activated carbon attained 22.8. The review also pointed out challenges related to preparation cost and stability for various adsorbents. Zhang et al. [65] employed grand canonical Monte Carlo (GCMC) and molecular dynamics (MD) methods to construct models for three unconventional gas reservoirs (coalbed, shale, and tight sandstone), investigating the adsorption mechanisms during CH_4_ displacement by CO_2_-rich industrial waste gas (containing SO_2_, NO, N_2_, etc.). The experimental results showed that across all reservoirs, the order of adsorption energy between CH_4_ and waste gas components was CH_4_/SO_2_ > CH_4_/CO_2_ > CH_4_/NO > CH_4_/N_2_. In coal seams, the absolute adsorption energy for CH_4_/SO_2_ was as high as 64.220 kJ/mol. At depths of 0.5–4.0 km, the adsorption selectivity of SO_2_, CO_2_, and NO over CH_4_ was greater than 1.0. In tight sandstone, gas adsorption was dominated by pore filling, while in coal seams and shale it was highly dependent on adsorption sites. Furthermore, competitive adsorption in deep reservoirs was found to adversely affect separation efficiency.

To accelerate the industrialization process of membrane technology, Su et al. [66] synthesized a cost-effective zinc-based metal organic framework material, CALF-20, conducting research through preparation and characterization, adsorption testing, molecular simulation, breakthrough experiments, and stability and cost analysis, focusing on the methane/nitrogen system. At 298 K and 0.5 kPa, its CH_4_/N_2_ selectivity reached 15.0, and at 100 kPa, its CH_4_ adsorption capacity was 1.11 mmol/g. Breakthrough experiments confirmed its effectiveness in separating CH_4_/N_2_ mixtures (1/1, *v*/*v*), and water vapor did not affect separation performance. Molecular simulation indicated that more binding sites and closer interaction distances between CH_4_ and the framework were key to the high selectivity. Simultaneously, this material exhibited thermal stability up to 613 K, excellent water and humidity stability, good cycling performance, and a ligand cost of only 53 USD/kg, making it a promising material for industrial CH_4_/N_2_ separation. Beyond the membrane technologies discussed above, utilizing adsorption separation technology for methane separation also holds significant potential. In this field, researchers mainly aim to improve CH_4_/N_2_ separation performance by controlling pore size, surface modification, and element doping. For example, Gu et al. [67] prepared GAC(C-12) via pre-oxidation/carbonization/steam activation, which showed a CH_4_ adsorption capacity of 2.3 mmol/g and selectivity of 3.17 at 298 K and 1 MPa. Further, Yao et al. [68] prepared CICTF-1-650 by pyrolysis at 650 °C, achieving a nitrogen content of 12 at%. Its CH_4_ adsorption capacity was 1.47 mmol/g and selectivity was 8.1, indicating that high nitrogen doping helps enhance CH_4_ selective adsorption. Additionally, Zhang et al. [69] prepared ACK2N1 material using non-corrosive potassium citrate and urea, achieving a selectivity of 7.11 and a CH_4_ adsorption capacity of 3.0 mmol/g at 273 K and 1 bar, demonstrating the potential of green synthesis. Notably, Chen et al. [70] prepared C-PVDC 700 material via activation-free pyrolysis, achieving a high selectivity of 14.7 and a CH_4_ adsorption capacity of 1.57 mmol/g at 298 K and 1 bar, indicating its advantage in high-selectivity CH_4_ capture.

Based on the multidimensional data analysis and comprehensive considerations above, Membrane technology demonstrates considerable potential across three main application areas in methane separation. For biogas upgrading, process optimization and the use of anti-plasticization membrane materials have enabled efficient methane recovery, with novel composite membranes further enhancing separation performance [71]. In natural gas decarbonization, the modular nature of membrane systems makes them suitable for high-pressure, high-CO_2_ environments, where material design and integrated processes improve techno-economic feasibility [72]. For unconventional natural gas (N_2_/CH_4_ separation), precisely tailored blend membranes and mixed-matrix membranes have significantly boosted selectivity [73]. However, challenges remain regarding material stability, cost control, and adaptability to real operating conditions, necessitating coordinated innovation in both materials and process engineering to advance practical implementation [74]. In addition to the application scenarios discussed above, hydrogen/methane (H_2_/CH_4_) separation holds significant potential in hydrogen production from natural gas reforming, coke oven gas purification, and the broader hydrogen energy chain. Although the kinetic diameter of H_2_ (0.29 nm) is considerably smaller than that of CH_4_ (0.38 nm), which theoretically favors molecular sieving, practical applications require a balance between high permeability and high selectivity. Several membrane materials reviewed in this paper have demonstrated promise in this context. For instance, Cai et al. developed carbon molecular sieve membranes with tailored precursor structures, achieving a H_2_/CH_4_ selectivity as high as 610. Lai et al. synthesized hydrocarbon ladder polymers that, after physical aging, exhibited a H_2_/CH_4_ selectivity of 621 while maintaining high permeability. Although Zhou et al. focused on N_2_/CH_4_ separation using MOF membranes with asymmetric pore window engineering, their pore architecture design strategy offers valuable insights for H_2_/CH_4_ separation. These findings suggest that existing membrane material systems, through precise pore structure regulation, can be extended to H_2_/CH_4_ separation. Future efforts should combine molecular simulation with materials design to further explore their potential in hydrogen-related applications. The following is a summary table that presents a comparison of the properties of different materials, as detailed in Table 2.

## 4. Discussion

Based on the review presented in this paper, although current methane separation membrane technologies have achieved significant advancements in areas such as the thermal cross-linking modification of polymeric membranes, MOF-based mixed matrix membranes, and carbon molecular sieve membranes [75], their large-scale application remains constrained by bottlenecks including insufficient material anti-plasticization properties [76], filler-matrix interfacial defects, and high-cost manufacturing [77]. Future research should focus on developing novel materials, such as MOF glasses, that combine high stability with precise molecular sieving functionality, and address interfacial compatibility issues through surface modification and process optimization [78,79]. Concurrently, efforts should integrate process intensification and intelligent control [80] to promote the efficient and stable operation of membrane technology in practical applications such as biogas upgrading and natural gas decarbonization [81,82], thereby providing reliable technical support for methane valorization and the low-carbon transformation of the energy structure [83].

For practical industrial applications, the long-term stability and tolerance of membrane materials are critical bottlenecks that urgently need to be addressed. Firstly, plasticization resistance is key in high-pressure natural gas purification. Plasticization arises from the dissolution of high-pressure CO_2_ or heavy hydrocarbons into the polymer matrix, which increases chain segment mobility and free volume, leading to a sharp decline in selectivity. Current mainstream anti-plasticization strategies include the following: (i) Chemical cross-linking, which restricts chain movement by introducing covalent bonds, such as in thermally cross-linked or UV-cross-linked polyimides [84]; (ii) Constructing rigid chain structures, such as using ladder or semi-ladder polymers whose inherent rigidity effectively resists swelling. Secondly, impurity resistance is particularly crucial in the treatment of landfill gas or coalbed methane. Trace amounts of H_2_S, water vapor, or heavy hydrocarbons in the feed gas can cause membrane poisoning, hydrolysis, or pore blockage. To address this issue, researchers are exploring: (i) Surface coating modification, such as coating with a hydrophilic polydopamine layer to prevent the adsorption of hydrophobic pollutants; (ii) Developing inherently impurity-tolerant inorganic membranes, such as zeolite molecular sieve membranes and MOF glass membranes, whose inorganic frameworks exhibit excellent chemical stability and anti-fouling capabilities [85]. These stability-oriented designs are essential pathways for advancing membrane technology from the laboratory to industrial sites.

## 5. Conclusions

As an advanced separation technology, membrane technology is playing an increasingly important role in the separation and purification of methane-containing gases. This paper systematically reviews the research from the aspects of basic principles, performance evaluation, material systems, and application status. Currently, new-generation membrane materials represented by mixed matrix membranes are continuously breaking through traditional performance upper bounds. In particular, the introduction of MOFs and their derivatives (such as MOF glasses) provides unprecedented opportunities for designing high-performance separation membranes. The application of this technology in areas such as biogas upgrading and natural gas decarbonation has proven its technical feasibility and economic potential [86]. However, the technology still faces challenges such as long-term material stability, large-scale manufacturing costs, and adaptability to complex operating conditions. Future research should focus on creating new materials, solving interface problems, innovating preparation processes, and optimizing integrated system processes. It is foreseeable that with the continuous advancement of materials science and chemical engineering, membrane technology will undoubtedly contribute key power in achieving utilization, promoting energy structure transformation, and addressing climate change [87,88].

## Figures and Tables

**Table 1:** Summary of Membrane Performance in CH_4_-Selective Separation.

Membrane Type	Material/System	Permeability P (Barrer) or Adsorption Capacity	Selectivity α
Polymeric	6FDA-Durene/CARDO(OH)	P(CO_2_) = 219	α(CO_2_/CH_4_) = 26.1
	SBS21	P(CH_4_) = 289	α(CH_4_/N_2_) = 7.2
	SBS-PDMS-co-PMHS (70%)	P(CH_4_) = 443.6	α(CH_4_/N_2_) = 3.10
	Pentiptycene P1	P(CO_2_) = 220	α(CO_2_/CH_4_) = 21.41
	Benzotriptycene-based PIMs (PIM-BTrip)	P(CO_2_) = 3770–4900	α(CO_2_/CH_4_) = 26.0–33.5
	3D CANAL ladder polymers (CANAL-Me-DHP)	P(CO_2_) = 94; P(H_2_) = 860	α(CO_2_/CH_4_) = 68; α(H_2_/CH_4_) = 621
Inorganic	TR-CMS-0.5	–	α(H_2_/CH_4_) = 610, α(CO_2_/CH_4_) = 143
	IL/CMS(Ionic Liquid Composite)	P(CO_2_) > 600	α(CO_2_/N_2_) > 50
	NCHA(Nano K-Chabazite)	Q(CH_4_) = 40.12 cm^3^/g	α(CH_4_/N_2_) = 4.7
	Zr-fum_67_-mes_33_-fcu-MOF	P(N_2_) = 3057 GPU	α(N_2_/CH_4_) = 15
Mixed Matrix	UiO-66/6FDA Polyimide Blend	P(CO_2_) increased by 635%	Selectivity maintained
	ZIF-301/6FDA-DAM	P(CO_2_) = 891	α(CO_2_/CH_4_) = 29.3
	Zeolite 4A (20%)/Polyetherimide	P(CO_2_) increased >90%	α(CO_2_/CH_4_) = 3.244
	POPs/CS:PVA (5%)	–	α(CO_2_/CH_4_) = 66.59
	AlFFIVE-1-Ni (001) nanosheets/6FDA-DAM	P(CO_2_) = ~2035 (predicted)	α(CO_2_/CH_4_) = 354 (predicted)
	Na-SSZ-39 (50 wt%)/Matrimid	P(CO_2_) = ~8280	α(CO_2_/CH_4_) = 423

Notes: P: Permeability (Barrer). Q: Adsorption capacity (cm^3^/g, STP). α(A/B): Selectivity of component A over B. GPU: Gas Permeation Unit (1 GPU = 3.35 × 10^−10^ mol·m^−2^·s^−1^·Pa^−1^).

**Table 2:** Performance Summary of Membrane Technology in CH_4_ Separation Applications.

Application Scenario	Material/Process System	Key Performance Metrics	Selectivity or Efficiency
Biogas Upgrading (CO_2_/CH_4_)	PI/Ionic Liquid (IL_3_, 15 wt%)	P(CO_2_) = 16.25 Barrer	α(CO_2_/CH_4_) = 180.55
	SERAN Membrane Module (Industrial)	CH_4_ purity: 96.99%	CO_2_ content reduced to 1.84%; CH_4_ recovery: 96.2%
Natural Gas Decarbonization (CO_2_/CH_4_)	COF-5 Membrane (Simulation)	Adsorption selectivity ratio: 17.46	High CO_2_/CH_4_ selectivity
	Graphene/SiC/BN Nanochannel	Pressure-dependent permeability	Tunable CH_4_/CO_2_ separation
	Integrated Membrane-LNG Process	Feed CO_2_: 46.7%; LNG CH_4_: 87.97%	Processing scale: 130,000 m^3^/d (decarbonation) + 100 t/h (LNG)
Unconventional NG Treatment (N_2_/CH_4_)	Pebax/EVA Blend (30 wt%)	P(CH_4_) = 384.6 Barrer	–
	PIM-1/CuBTC MMMs	P(CH_4_) = 8120 Barrer	α(CH_4_/N_2_) = 6.52
	Zn-based MOF (CALF-20)	Q(CH_4_) = 1.11 mmol/g	α(CH_4_/N_2_) = 15.0
	GAC(C-12) Activated Carbon	Q(CH_4_) = 2.3 mmol/g (1 MPa)	α(CH_4_/N_2_) = 3.17
	CICTF-1-650 (N-doped Carbon)	Q(CH_4_) = 1.47 mmol/g	α(CH_4_/N_2_) = 8.1
	ACK2N1 (Green Synthesis)	Q(CH_4_) = 3.0 mmol/g (273 K)	α(CH_4_/N_2_) = 7.11
	C-PVDC 700 (Activation-free)	Q(CH_4_) = 1.57 mmol/g	α(CH_4_/N_2_) = 14.7

Notes: P: Permeability (Barrer). Q: Equilibrium adsorption capacity (mmol/g). α(A/B): Selectivity of component A over B.

## Data Availability

No new data were created or analyzed in this study. Data sharing is not applicable to this article.
